# Recommendations for Assessment of the Reliability, Sensitivity, and Validity of Data Provided by Wearable Sensors Designed for Monitoring Physical Activity

**DOI:** 10.2196/mhealth.9341

**Published:** 2018-04-30

**Authors:** Peter Düking, Franz Konstantin Fuss, Hans-Christer Holmberg, Billy Sperlich

**Affiliations:** ^1^ Integrative & Experimental Exercise Science & Training Institute for Sport Sciences University of Würzburg Würzburg Germany; ^2^ Swedish Winter Sports Research Centre Mid Sweden University Östersund Sweden; ^3^ Smart Equipment Engineering and Wearable Technology Research Program Centre for Design Innovation Swinburne University of Technology Melbourne Australia; ^4^ School of Sport Sciences University of Tromsø - The Arctic University of Norway Tromsø Norway; ^5^ School of Kinesiology University of British Columbia Vancouver, BC Canada

**Keywords:** activity tracker, data mining, Internet of Things, load management, physical activity, smartwatch

## Abstract

Although it is becoming increasingly popular to monitor parameters related to training, recovery, and health with wearable sensor technology (wearables), scientific evaluation of the reliability, sensitivity, and validity of such data is limited and, where available, has involved a wide variety of approaches. To improve the trustworthiness of data collected by wearables and facilitate comparisons, we have outlined recommendations for standardized evaluation. We discuss the wearable devices themselves, as well as experimental and statistical considerations. Adherence to these recommendations should be beneficial not only for the individual, but also for regulatory organizations and insurance companies.

## Introduction

Wearable sensors (so-called “wearables”) are currently the world’s leading trend in fitness [1,2] and are being employed widely by various groups to monitor variables related to health, physical activity, training load, and recovery [3,4], often with the goal of individualizing physical activity and improving performance. Several insurance companies promote such monitoring [5] and an increasing number of organizations that regulate sports (eg, the International Football Association Board [6]), allow wearables to be worn during competitions (albeit with certain limitations).

If wearables are to be of value in enhancing health and performance [4], it is becoming more and more imperative that the data they supply are proven to be trustworthy by employing scientific approaches [7]. Unfortunately, wearables are often marketed with aggressive and exaggerated claims that lack a sound scientific basis [7], and the unreliable data they provide (and/or interpretation thereof) has resulted in costly class-action lawsuits [8] and provides little or no value to the customer.

Recent scientific evaluation of wearable data has involved widely heterogeneous study designs (including the nature and size of the study population), methodologies, criteria for comparisons, terminologies, and statistical analyses, as well as varying intensities/modalities of exercise. Assessment of novel technology may be influenced by the particular test conditions employed [9]. For example, laboratory data may not be transferable to real-life situations and data trustworthy in a resting condition or during low-intensity exercise may become less valid at higher intensity (eg, due to motion artifacts). Thus, variations in methodology complicate the comparison of scientific evaluations of wearable data.

From our perspective, athletes, manufacturers of wearables, and organizations concerned with health, sports, and insurance could all benefit from basic recommendations for assessment of the reliability, sensitivity, and validity of data provided by wearable sensors. The aim of this paper is to formulate such recommendations.

## Factors Inherent to the Wearables Themselves

### Sensor Characteristics

Wearables contain a wide variety of sensors (eg, electrochemical, optical, acoustic, and/or pressure‑sensitive), as well as inertial measurement units and global navigation satellite systems (including global positioning systems [GPS]). More than one of these are often present within the same device. These sensors, produced by various manufacturers, are designed to monitor a variety of internal (eg, heart rate, tissue oxygenation, distribution of plantar pressure) and/or external (eg, acceleration of body segments, speed while exercising) parameters, mostly noninvasively [3]. With multi‑sensor devices, the quality of data and parameters derived depends on the interplay between the sensors, each of which must therefore be scrutinized both independently and in combination with the others. Consideration of individual sensors is beyond the scope of the present recommendations and we refer the reader to other relevant work for such information [3,10,11].

### Software

The nature of the software in the wearable itself, as well as of the software in any accompanying device (ie, laptop, smartphone application) exerts a considerable influence on data quality. For example, the software in GPS receivers or analytical software on an accompanying device may actually alter data [12-14]. We therefore urge researchers to describe the software utilized by the wearable and accompanying devices and/or the involvement of “cloud” technology in detail.

### Acquisition of Raw Data: Sampling Frequency and Filtering

Although of less concern to the private consumer, to improve the reliability, sensitivity, and validity of data used for research purposes we recommend that manufacturers provide access to raw data. This issue is of particular interest in the case of multi-sensor devices, which often calculate a single value by combining data from several sensors (a common example being calculation of energy expenditure by merging heart rate with several GPS parameters), yet the contribution by each individual sensor is often unclear. Describing these contributions could enhance scientific trustworthiness (eg, by improving the algorithms employed).

A high sampling frequency, which normally enhances data quality, may be achieved artificially by filtering techniques (eg, interpolation) that produce no actual improvement in this quality [15]. Consequently, both the sampling frequency and any filtering techniques applied should be described in detail.

### Durability

Sensors can deteriorate or even wear out with extended use and it is clearly important to describe the durability of the wearable and its sensor technology, at least as indicated by the manufacturer. Unlike laboratory equipment, most wearables are not checked routinely, making such description essential. Wearable devices are typically brand-new when evaluated and the quality and trustworthiness of the data they provide may change with use.

### Precise Reporting of Anatomical Positioning

Wearables and their algorithms are often designed for use at a specific position or region of the body, which, consequently, must be indicated clearly. In certain cases, imprecise positioning may attenuate data quality [3]. For example, sensors for surface electromyography incorporated into clothing must be positioned precisely on the muscle, preferably along the midline, halfway between the entrance of the nerve and myotendinous junction [16]. On a daily basis, such accurate positioning may prove difficult, especially since this is often performed by nonprofessionals. Moreover, signal reproducibility may be affected by repeated donning and removal of garments. Consequently, we encourage researchers to describe in detail the positioning of wearables, as well as reproducibility of data. Researchers often evaluate several wearables simultaneously and such devices in close proximity can interfere with one another [15]. We recommend strongly that any potential interference be controlled for.

## Experimental Considerations

### Study Population

Selection of the study population (eg, cyclists, runners or team members, elite or recreational athletes, youth or adults, men or women) should accurately reflect the intended use of the wearable. Each population behaves differently (eg, with respect to lifestyle) and algorithms should be transferred from one specific population to another only with great care. The inclusion and exclusion criteria for participants must be described clearly. If anyone opts out of the experimental procedure or data analysis, a reason should be given.

### Exercise Protocol

The intended purpose and conditions for use of the wearable should be clarified. If designed for monitoring general activity, data should be collected in connection with various forms of exercise (eg, running, cycling, rowing, intermittent activities, activities of daily living) of varying intensity (eg, resting, submaximal, high), in different positions (lying, sitting, or standing), and/or while moving freely. If a wearable is intended to be used in connection with team sports such as soccer, a protocol mimicking the demands of this sport–including low-speed running, straight sprints, change‑of‑direction, and tackling–is much more preferable than running constantly at low speed only.

### Potential Confounders

Factors that could influence the outcome, such as temperature and humidity, the warm‑up procedure, nutritional status, and any form of encouragement, should resemble the real‑life situation as closely as possible [17,18] and be described in detail.

Other potential confounders may also need to be taken into consideration. For example, sensors that monitor electrical signals (eg, for electromyography or electrocardiography) may be influenced by other devices, such as a participant’s pacemaker. Optical sensors (eg, for photoplethysmography) can be affected by the photosensitivity of the skin or by vasoconstriction [19,20]. In the case of GPS receivers, the horizontal dilution of precision, as well as the number of satellites to which the wearable is connected, should be reported [15]. Although there are no clear rules, two wearables should not be tested at the same time (eg, one on top of the other) or, if they are, potential interference and crosstalk should be examined for by switching the positions of the devices [21]. Adequate controlling for numerous confounding factors requires a good understanding of both the sensor technology and associated physiological and/or biomechanical processes.

### Special Considerations Concerning Reliability

Intradevice reliability concerns reproducibility within the same device [22,23], while interdevice reliability (reproducibility with different devices) is to be tested if the devices in question are intended for interchangeable use [12]. Both types of reliability should be confirmed routinely. Recently, it has been recommended that at least 50 participants and three trials should be involved in order to obtain precise estimates of reliability [23]. When multiple trials are performed at different times, potential confounders must vary as little as possible.

### Special Considerations Concerning Validity

Several different types of validity (eg, logical, convergent, and construct validity [24,25]) are probably equally important in this context, but discussion of these in detail is beyond the present scope and we refer the interested reader to other relevant articles [24,25]. Here, we focus on concurrent criterion validity, since this is probably easiest to access with respect to wearables. Concurrent criterion validity evaluates the association between data provided by the new device and another device considered to be more valid (sometimes referred to as a criterion measure or “gold‑standard”) [23,25].

For certain parameters, there are generally-accepted criterion measures (eg, polysomnographic parameters of sleep [26] and an ingestible telemetric sensor for core body temperature [27]). However, for others (eg, energy expenditure at several timepoints while moving freely and in-shoe plantar pressure) no such measures are currently available. We encourage researchers to describe the trustworthiness of their criterion measures and strongly discourage the use of measures not considered to be “gold‑standard” for validation of the quality of wearable data.

## Statistical Analyses

### Overview

The various statistical approaches for evaluating the reliability or validity of wearables all have limitations [28,29]. Without discouraging the usage of other robust approaches (eg, the Standard Error of Measurement for reliability studies [28] or Bland‑Altman plots for validity studies [30,31]), we propose one possible approach to statistical assessment of wearable data concerning reliability, sensitivity, and validity in the following sections.

### Reliability

Reliability should be documented in terms of intrasubject variability (eg, measured as standard deviation, “...the random variation in a measure when one individual is tested many times”), which is possibly the most important indicator of the reliability of measures of performance and sometimes referred to as typical error (TE) [23]. The TE can also be expressed as the coefficient of variation (%CV) [23] and we encourage the reporting of both.

Another measure of reliability (eg, “...the change in mean value between 2 trials...”) assesses systematic bias in combination with random variations [23]. The random variation is simply a sampling error, which tends to be smaller with larger samples. Systematic bias can be due to learning by (and training of) subjects or effects related to fatigue, and consequently can often be minimized by familiarization trials or adequate rest between trials, respectively [23].

In addition, researchers should assess test-retest reliability with the intraclass correlation coefficient [32], which “represents how closely the values of one trial track the values of another as we move our attention from individual to individual” [23] or, in other words, the reliability “of the position or rank of individuals in the group relative to others” [28]. Moreover, to determine whether data provided by different wearables can be used interchangeably, it may be of interest to evaluate interdevice reliability, previously accomplished by calculating the %CV between the devices when worn simultaneously [12].

### Sensitivity

Wearables designed to track changes in performance and/or parameters over time must, of course, be sensitive to such changes [33]. Even with a reliable test, the noise can be high enough to mask changes in parameters [33]. In the case of individual elite athletes, for whom certain fitness parameters are directly correlated with performance (eg, energy expenditure at a given running intensity; the lower, the less intense), the smallest worthwhile change (SWC) is 30% of the individual’s typical variation in performance [34]. Where there is no clear relationship between parameters of fitness and performance (eg, strength and team sport performance), it has been proposed that the SWC be calculated (0.2 times the between-subject standard deviation, based on Cohen’s effect size principle) and compared with the noise of the measuring device or test [33,34]. This noise can be expressed as the TE, which can be obtained from reliability studies, as described above. A TE less than, similar to, or higher than the SWC can be rated as “good,” “OK,” or “marginal,” respectively [33]. When assessing sensitivity, similar and reliable experimental approaches are required.

### Validity

Linear regression analysis can be employed to identify bias and provide an estimate of the TE in wearable data [29,35,36]. Furthermore, Pearson’s product‑moment correlation coefficient should be calculated [36] to compare the degree of association [33,37] between data obtained with the criterion measure and the wearable. However, a significant correlation does not definitively mean that these data do not differ and is not, therefore, on its own a sufficient indicator of validity [30].

## Conclusions

Here, we have outlined general recommendations (summarized in Table 1) for the evaluation of the trustworthiness of monitoring training load, recovery, and health by wearables. We are well aware that with certain technologies, other methodological considerations may be of particular importance and that new approaches are emerging constantly. Although evaluation may not be possible or even desirable in every individual context, findings in one situation should be transferred to another only with great care and appropriate justification.

The market for wearables is growing exponentially and their scientific evaluation in a trustworthy manner needs to keep pace. The success of a wearable device depends on gaining the trust of the consumer, stakeholders, and policymakers alike (eg, by transparent reporting of standardized validation, ideally carried out by an independent research institution). We are convinced that these recommendations can aid manufacturers of wearables, athletes, coaches, team managers, insurance companies, and other stakeholders and policymakers alike in evaluating wearable sensor technologies and/or selecting appropriate devices.

**Table 1:** Checklist of important considerations associated with the evaluation of data provided by wearables.

Factor	Action/recommendation
Sensor characteristics	Scrutiny of each sensor
Software	Specify calculations/algorithms Report the version of software and firmware involved
Raw data	Report sampling frequency Report filtering techniques and aggregation
Durability	Report the durability and age of the device
Anatomical positioning	Report the precise anatomical positioning of sensors Report signal reproducibility upon repeated putting on and taking off Report considerations concerning positioning Control for and describe potential interference
Study population	Describe the target population Specify inclusion and exclusion criteria Generalize to other populations only with great care
Exercise protocol	Describe conditions (eg, ambient temperature, altitude) in as much detail as possible Investigate different forms of exercise (running, cycling, walking, moving freely) Apply different intensities (lying, sitting, low and high intensity)
Confounders	Report any potential confounding factors Perform assessment in both controlled and real-life scenarios Check for potential crosstalk between devices
Assessment of reliability	Determine intradevice and interdevice reliability Document intrasubject standard deviation Report the coefficient of variation Calculate the intraclass correlation coefficient Recruit at least 50 participants Report systematic bias
Assessment of sensitivity	Calculate the smallest worthwhile change
Assessment of validity	Choose an appropriate criterion measure and assess the reliability of this measure as well Perform linear regression analysis Calculate Pearson’s product-moment correlation

## Abbreviations

%CV: coefficient of variation
GPS: global positioning system
SWC: smallest worthwhile change
TE: typical error
